# ﻿Complete mitogenome of the critically endangered Asian king vulture (*Sarcogypscalvus*) (Aves, Accipitriformes, Accipitridae): evolutionary insights and comparative analysis

**DOI:** 10.3897/zookeys.1234.138722

**Published:** 2025-04-08

**Authors:** Wannapol Buthasane, Sithichoke Tangphatsornruang, Piroon Jenjaroenpun, Thidathip Wongsurawat, Saowaphang Sanannu, Vorasuk Shotelersuk, Gunnaporn Suriyaphol

**Affiliations:** 1 Biochemistry Unit, Department of Physiology, Faculty of Veterinary Science, Chulalongkorn University, Bangkok 10330, Thailand; 2 National Omics Center, National Center for Genetic Engineering and Biotechnology (BIOTEC), National Science and Technology Development Agency, Pathum Thani 12120, Thailand; 3 Division of Medical Bioinformatics, Research Department, Faculty of Medicine Siriraj Hospital, Mahidol University, Bangkok 10700, Thailand; 4 Animal Conservation and Research Institute, The Zoological Park Organization of Thailand, Bangkok 10300, Thailand; 5 Center of Excellence for Medical Genomics, Medical Genomics Cluster, Department of Pediatrics, Faculty of Medicine, Chulalongkorn University, Bangkok 10330, Thailand; 6 Excellence Center for Genomics and Precision Medicine, King Chulalongkorn Memorial Hospital, The Thai Red Cross Society, Bangkok 10330, Thailand

**Keywords:** Asian king vulture, comparative analysis, evolution, mitogenome, *Sarcogypscalvus*

## Abstract

The Asian king vulture (*Sarcogypscalvus*), also known as the red-headed vulture, is an Old World vulture (Gypini) facing severe population declines. This study aimed to assemble the complete mitogenome of *S.calvus*, explore its phylogenetic relationships, estimate divergence times, and examine genetic distances and amino acid substitutions. The mitogenome was de novo assembled from genomic DNA extracted from the blood of a female *S.calvus*. Phylogenetic and pairwise genetic distance analyses were conducted, comparing *S.calvus* with other members of Gypini, New World vultures (Cathartidae) and various other birds. The assembled mitogenome was 17,750 base pairs in length, comprising 13 protein-coding genes (PCGs), 22 transfer RNA genes, two ribosomal RNA genes and two control regions. Most PCGs used the ATG start codon, except for cytochrome c oxidase subunit 1 (*COX1*), which employed GTG. Phylogenetic analysis revealed a close genetic relationship between *S.calvus* and other members of Gypini, with an estimated divergence time of 16.7 million years ago. Genetic distance analysis indicated that *S.calvus* was more closely related to other Gypini, as well as to *Spilornischeela* and *Circaetuspectoralis* (Circaetini)), than to Cathartidae. Conserved amino acid substitutions between Gypini and Cathartidae were primarily observed in the NADH-ubiquinone oxidoreductase chain 1 (*ND1*) gene. This study provided the first complete mitogenome of *S.calvus*, offering new insights into its genomic structure, evolutionary history, and genetic relationships.

## ﻿Introduction

The Asian king vulture (*Sarcogypscalvus* Scopoli, 1786), also known as the red-headed vulture, belongs to the Old World vulture group (tribe Gypini) within the order Accipitriformes and the family Accipitridae. Classified as Critically Endangered by the International Union for Conservation of Nature (IUCN) Red List of Threatened Species, *S.calvus* is also listed under Appendix II of the Convention on International Trade in Endangered Species of Wild Fauna and Flora (CITES) (BirdLife International 2021; CITES 2024). Additionally, the species is protected under Thailand’s Wild Animal Conservation and Protection Act, B.E. 2562 (2019) (FAOLEX Database 2019). Gypini are distributed across Africa, Asia, and Europe, while New World vultures (Cathartidae) inhabit the Americas. Currently, the global population of *S.calvus* in the wild is estimated to be between 2,500 and 9,999 individuals, while in Thailand, the species is possibly extinct in the wild and only seven individuals remain in captivity (BirdLife International 2021; Buthasane et al. 2024).

The mitogenome (mitochondrial genome) is a valuable tool for investigating phylogenetic relationships, molecular identification, and adaptive evolution (De Panis et al. 2021; Kong et al. 2024). Mitogenomes of four species of Gypini have been reported, i.e., those of the Eurasian griffon (*Gypsfulvus* Hablizl, 1783), cape vulture (*Gypscoprotheres* Forster, 1798), cinereous vulture (*Aegypiusmonachus* Linnaeus, 1766) and Himalayan griffon (*Gypshimalayensis* Hume, 1869) (Li et al. 2015; Mereu et al. 2017; Jiang et al. 2019; Adawaren et al. 2020). For Cathartidae, seven mitogenomes from six species have been documented, i.e. the Andean condor (*Vulturgryphus* Linnaeus, 1758), king vulture (*Sarcoramphuspapa* Linnaeus, 1758), California condor (*Gymnogypscalifornianus* Shaw, 1797), lesser yellow-headed vulture (*Cathartesburrovianus* Cassin, 1845), turkey vulture (*Cathartesaura* Linnaeus, 1758) and black vulture (*Coragypsatratus* Bechstein, 1793) (Slack et al. 2007; De Panis et al. 2021; Urantówka et al. 2021). In addition, 11 mitogenomes from other species in the family Accipitridae have been reported, including those of the golden eagle (*Aquilachrysaetos* Linnaeus, 1758), common buzzard (*Buteobuteo* Linnaeus, 1758) and black kite (*Milvusmigrans* Boddaert, 1783) (Haring et al. 2001; Jeon et al. 2018; Mead et al. 2021). The genome of *S.calvus* has recently been published (Buthasane et al. 2024). However, no mitogenomic data are currently available for *S.calvus*, and its mitochondrial features remain understudied. This study aimed to elucidate the complete mitogenome of *S.calvus* and provide a comprehensive analysis of its structure, phylogenetic position, and the divergence time from other vultures. This research offers valuable insights into the mitochondrial profiles, evolutionary relationships, and population genetics of *S.calvus* in relation to other Gypini, Cathartidae and related species.

## ﻿Materials and methods

Whole blood samples were obtained from a female *S.calvus*, approximately 25 years old, at Nakhon Ratchasima Zoo, the Zoological Park Organization of Thailand (ZPOT). Sampling was conducted in compliance with the ethical guidelines under the Chulalongkorn University Animal Care and Use Committee (CU-ACUC), Thailand (approval number 2131005). Total DNA was extracted from the whole blood sample using the Wizard HMW DNA Extraction Kit (Promega, Madison, WI, USA). The DNA concentration was determined using a NanoDrop One Microvolume UV-Vis Spectrophotometer (Thermo Fisher Scientific, Waltham, MA, USA).

The mitochondrial genome was sequenced using the short-read MGISEQ-2000 platform (MGI Tech, Shenzhen, China) and assembled with NOVOPlasty v. 3.8.2 (Dierckxsens et al. 2017). Annotation was carried out using the MITOS WebServer (Bernt et al. 2013). Protein-coding, rRNA and tRNA genes were further identified using the NCBI Basic Local Alignment Search Tool (BLAST) (Altschul et al. 1990). The circular structure of the mitogenome was visualized with OrganellarGenomeDRAW (OGDRAW) v. 1.3.122 (Greiner et al. 2019). Analyses of nucleotide and amino acid composition were conducted using MEGA X (Kumar et al. 2018). Simple sequence repeats (SSRs) of 1–6 bp in length were identified using the Microsatellite identification tool (Beier et al. 2017).

Mitogenomes from 39 bird species, representing the orders Accipitriformes (Old World vultures, hawks, eagles, and kites), Cathartiformes (New World vultures), Falconiformes (falcons), Strigiformes (owls), Anseriformes (ducks and relatives) and Galliformes (chickens and relatives), were used for phylogeny reconstruction, comparative codon usage analyses (Table 1) and divergence time estimation. These mitogenomes, along with the newly sequenced mitogenome of *S.calvus*, were analyzed. Multiple sequence alignments of 13 conserved protein-coding genes (PCGs)−cytochrome B (*CYTB*), NADH dehydrogenase subunits 1 (*ND1*), *ND2*, *ND3*, *ND4*, *ND4L*, *ND5*, *ND6*, cytochrome c oxidase subunits 1 (*COX1*), *COX2*, *COX3*, ATP synthase F0 subunit 6 (*ATP6*) and *ATP8*−were performed using PRANK v170427. The best-fit model, mtVer+I+R4, was selected using ModelFinder, and maximum likelihood phylogenies were constructed using IQ-TREE v. 2.2.0.3 with 1,000 ultrafast bootstrap replications (Nguyen LT et al. 2014; Hoang et al. 2017). The phylogenetic tree was visualized with Figtree v. 1.4.4 (Rambaut 2018). Several species of Anseriformes and Galliformes were used as outgroups. Divergence times were estimated using MCMCTree in the PAML 4.9j package, with the Hessian matrix computed via CODEML and a burn-in of 2,000 iterations. Fossil calibration times were obtained from the TimeTree database (Kumar et al. 2022). Genetic distance analyses were conducted using MEGA X and visualized using ggplot2 and ggtree in R (Wickham 2016; Yu et al. 2017; Kumar et al. 2018). The nomenclature for higher taxa follows Gregory et al. (2024).

**Table 1.** List of 39 avian species employed for comparative mitogenome analyses in this study, along with their corresponding GenBank accession numbers.

Scientific name	Order	Family	Accesion number	Sequence length (bp)	Reference
*Accipitergentilis*	Accipitriformes	Accipitridae	NC_011818.1	18,266	Unpublished
*Accipitervirgatus*	Accipitriformes	Accipitridae	NC_026082.1	17,952	Song et al. 2015
*Aegypiusmonachus*	Accipitriformes	Accipitridae	KF682364.1	17,811	Li et al. 2015
*Aquilachrysaetos*	Accipitriformes	Accipitridae	NC_024087.1	17,332	Doyle et al. 2014
*Buteobuteo*	Accipitriformes	Accipitridae	NC_003128.3	18,674	Haring et al. 2001
*Circaetuspectoralis*	Accipitriformes	Accipitridae	NC_052805.1	17,473	Feng et al. 2020
*Circusmelanoleucos*	Accipitriformes	Accipitridae	NC_035801.1	17,749	Unpublished
*Gypscoprotheres*	Accipitriformes	Accipitridae	MF683387.1	16,908	Adawaren et al. 2020
*Gypsfulvus*	Accipitriformes	Accipitridae	NC_036050.1	18,094	Mereu et al. 2017
*Gypshimalayensis*	Accipitriformes	Accipitridae	KY594709.1	17,381	Jiang et al. 2019
*Haliaeetusalbicilla*	Accipitriformes	Accipitridae	NC_040858.1	17,719	Kim et al. 2019
*Haliasturindus*	Accipitriformes	Accipitridae	NC_066800.1	19,055	Sonongbua et al. 2024
*Milvusmigrans*	Accipitriformes	Accipitridae	NC_038195.1	18,016	Jeon et al. 2018
*Spilornischeela*	Accipitriformes	Accipitridae	NC_015887.1	18,291	Unpublished
*Spizaetustyrannus*	Accipitriformes	Accipitridae	NC_052803.1	17,479	Feng et al. 2020
*Pandionhaliaetus*	Accipitriformes	Pandionidae	NC_008550.1	19,285	Gibb et al. 2007
*Sagittariusserpentarius*	Accipitriformes	Sagittariidae	NC_023788.1	19,329	Mahmood et al. 2014
*Ansercygnoides*	Anseriformes	Anatidae	NC_023832.1	19,302	Mu et al. 2014
*Brantacanadensis*	Anseriformes	Anatidae	NC_007011.1	16,808	Snyder et al. 2015
*Anseranassemipalmata*	Anseriformes	Anseranatidae	NC_005933.1	16,870	Harrison et al. 2004
*Cathartesaura*	Cathartiformes	Cathartidae	NC_007628.1	16,870	Slack et al. 2007
*Cathartesburrovianus*	Cathartiformes	Cathartidae	NC_063526.1	16,779	Urantówka et al. 2021
*Coragypsatratus*	Cathartiformes	Cathartidae	NC_063525.1	17,864	Urantówka et al. 2021
*Gymnogypscalifornianus*	Cathartiformes	Cathartidae	BK059163.1	16,760	De Panis et al. 2021
*Sarcoramphuspapa*	Cathartiformes	Cathartidae	NC_063527.1	16,773	Urantówka et al. 2021
*Vulturgryphus*	Cathartiformes	Cathartidae	NC_058600.1	16,739	De Panis et al. 2021
*Caracaraplancus*	Falconiformes	Falconidae	NC_044672.1	17,077	Oswald et al. 2019
*Falcoperegrinus*	Falconiformes	Falconidae	NC_000878.1	18,068	Mindell et al. 1997
*Alecturalathami*	Galliformes	Megapodiidae	NC_007227.1	16,698	Slack et al. 2007
*Craxrubra*	Galliformes	Cracidae	NC_024618.1	16,666	Meiklejohn et al. 2014
*Callipeplasquamata*	Galliformes	Odontophoridae	NC_029340.1	16,701	Halley et al. 2015
*Gallusgallus*	Galliformes	Phasianidae	NC_053523.1	16,784	Unpublished
*Numidameleagris*	Galliformes	Numididae	NC_034374.1	16,785	Unpublished
*Asiootus*	Strigiformes	Strigidae	NC_039736.1	17,555	Lee et al. 2018
*Bubobubo*	Strigiformes	Strigidae	NC_038219.1	18,952	Kang et al. 2018
*Glaucidiumcuculoides*	Strigiformes	Strigidae	NC_034296.1	17,392	Unpublished
*Otussunia*	Strigiformes	Strigidae	NC_041422.1	17,835	Zhou et al. 2019
*Strixuralensis*	Strigiformes	Strigidae	NC_038218.1	18,708	Kang et al. 2018
*Phodilusbadius*	Strigiformes	Tytonidae	NC_023787.1	17,086	Mahmood et al. 2014

Relative synonymous codon usage (RSCU) values for *S.calvus* mitochondrial protein-coding genes were calculated using MEGA X (Kumar et al. 2018). RSCU values reflect codon bias, with values greater than one indicating positive codon bias, values less than one indicating a negative codon bias, and values equal to one indicating random codon usage (Wong et al. 2010).

Amino acid sequences from 13 mitochondrial protein-coding genes in the mitogenomes of Gypini (*Aegypiusmonachus*, *Gypscoprotheres*, *Gypsfulvus*, *Gypshimalayensis*, *S.calvus*) and Cathartidae (*Cathartesaura*, *Cathartesburrovianus*, *Coragypsatratus*, *Gymnogypscalifornianus*, *Sarcoramphuspapa*, *Vulturgryphus*) were aligned using the Unipro UGENE Multiple Alignment Editor (Okonechnikov et al. 2012).

## ﻿Results

**Figure 1.**
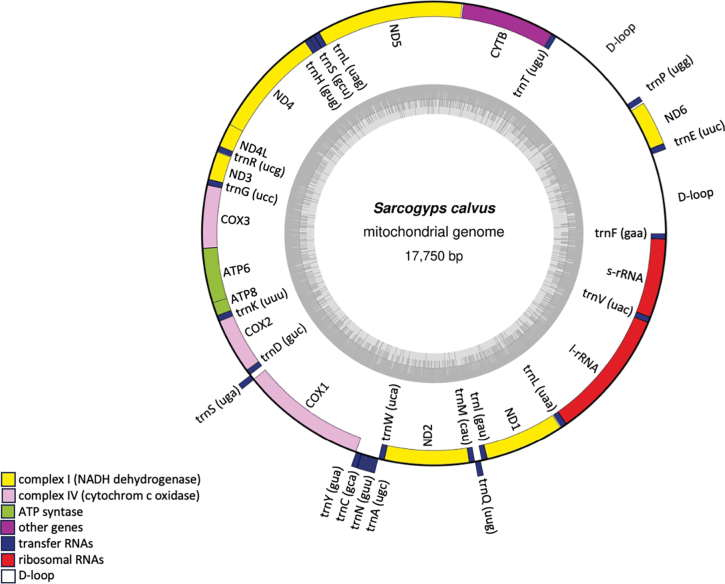
Circular mitogenome map of Asian king vulture. The complex I (NADH dehydrogenase), complex IV (cytochrome c oxidase), ATP synthase, ribosomal RNAs, transfer RNAs, cytochrome b and control region (D-loop) are annotated. Genes located outside the circle are transcribed in a clockwise direction, whilefig. genes inside are transcribed counterclockwise. The inner ring shadow denotes the GC content of the genome.

The complete mitogenome of *S.calvus* was determined to be 17,750 base pairs (bp) in length and was assigned GenBank accession number OR896160. The circular structure of the mitogenome of *S.calvus* is illustrated in Fig. 1. This mitogenome contained 13 PCGs, 22 transfer RNA genes (tRNAs), two ribosomal RNA genes and two putative control regions (CRs), also referred to as D-loop regions (Table 2). The nucleotide composition was characterized by 54.1% adenine and thymine (AT) and 45.9% guanine and cytosine (GC).

**Table 2.** Characteristics of the mitogenome of *Sarcogypscalvus*.

Start	End	Length (bp)	Direction	Type	Gene name	Gene product	Anti-codon	Start codon	Stop codon
1	987	987	+	control region	-	-	–	–	–
988	1058	71	+	tRNA	*trnE* (*uuc*)	tRNA-Glu	TTC	–	–
1059	1577	519	+	CDS	*ND6*	NADH dehydrogenase subunit 6	–	ATG	TAG
1599	1668	70	+	tRNA	*trnP* (*ugg*)	tRNA-Pro	TGG	–	–
1669	2863	1195	+	control region					
2931	2864	68	–	tRNA	*trnT* (*ugu*)	tRNA-Thr	TGT		
4076	2934	1143	–	CDS	*CYTB*	cytochrome b	–	ATG	TAA
5903	4089	1815	–	CDS	*ND5*	NADH dehydrogenase subunit 5	–	ATG	TAA
5974	5904	71	–	tRNA	*trnL* (*uag*)	tRNA-Leu	TAG	–	–
6039	5975	65	–	tRNA	*trnS* (*gcu*)	tRNA-Ser	GCT	–	–
6110	6041	70	–	tRNA	*trnH* (*gug*)	tRNA-His	GTG	–	–
7488	6106	1383	–	CDS	*ND4*	NADH dehydrogenase subunit 4	–	ATG	AGG
7778	7482	297	–	CDS	*ND4L*	NADH dehydrogenase subunit 4L	–	ATG	TAA
7848	7780	69	–	tRNA	*trnR* (*ucg*)	tRNA-Arg	TCG	–	–
8204	7854	354	–	CDS	*ND3*	NADH dehydrogenase subunit 3	–	ATG	AGG
8273	8205	69	–	tRNA	*trnG* (*ucc*)	tRNA-Gly	TCC	–	–
9057	8274	784	–	CDS	*COX3*	cytochrome c oxidase subunit III	–	ATG	CCT
9740	9057	684	–	CDS	*ATP6*	ATP synthase F0 subunit 6	–	ATG	TAA
9898	9731	168	–	CDS	*ATP8*	ATP synthase F0 subunit 8	–	ATG	TAA
9970	9900	71	–	tRNA	*trnK* (*uuu*)	tRNA-Lys	TTT	–	–
10655	9972	684	–	CDS	*COX2*	cytochrome c oxidase subunit II	–	ATG	TAA
10726	10658	69	–	tRNA	*trnD* (*guc*)	tRNA-Asp	GTC	–	–
10731	10802	72	+	tRNA	*trnS* (*uga*)	tRNA-Ser	TGA	–	–
12344	10794	1551	–	CDS	*COX1*	cytochrome c oxidase subunit I	–	GTG	AGG
12346	12415	70	+	tRNA	*trnY* (*gua*)	tRNA-Tyr	GTA	–	–
12416	12482	67	+	tRNA	*trnC* (*gca*)	tRNA-Cys	GCA	–	–
12485	12557	73	+	tRNA	*trnN* (*guu*)	tRNA-Asn	GTT	–	–
12560	12628	69	+	tRNA	*trnA* (*ugc*)	tRNA-Ala	TGC	–	–
12702	12630	73	–	tRNA	*trnW* (*uca*)	tRNA-Trp	TCA	–	–
13747	12701	1047	–	CDS	*ND2*	NADH dehydrogenase subunit 2	–	ATG	TAG
13816	13748	69	–	tRNA	*trnM* (*cau*)	tRNA-Met	CAT	–	–
13816	13886	71	+	tRNA	*trnQ* (*uug*)	tRNA-Gln	TTG	–	–
13971	13900	72	–	tRNA	*trnI* (*gau*)	tRNA-Ile	GAU	–	–
14947	13970	978	–	CDS	*ND1*	NADH dehydrogenase subunit 1	–	ATG	AGG
15030	14957	74	–	tRNA	*trnL* (*uaa*)	tRNA-Leu	TAA	–	–
16634	15030	1605	–	rRNA	*l*-*rRNA*	16S ribosomal RNA	–	–	–
16706	16635	72	–	tRNA	*trnV* (*uac*)	tRNA-Val	TAC	–	–
17681	16706	976	–	rRNA	*s-rRNA*	12S ribosomal RNA	–	–	–
17750	17681	70	–	tRNA	*trnF* (*gaa*)	tRNA-Phe	GAA	–	–

**Figure 2.**
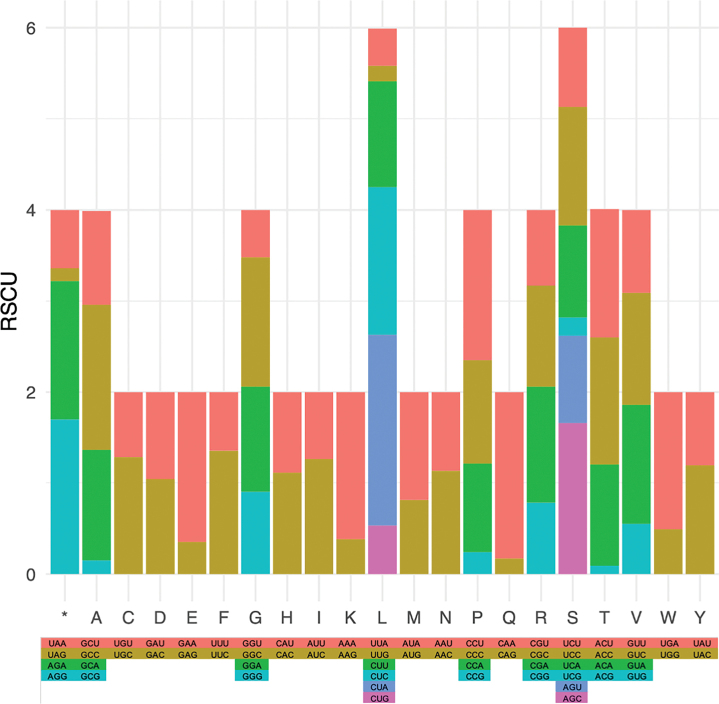
The relative synonymous codon usage (RSCU) and codon distribution of the Asian king vulture mitogenome. The different colors in the column chart symbolize distinct codon families associated with the amino acids listed below. Consistent coloring is applied to maintain representation uniformity across the same codon families. Bar chart showed-relative synonymous codon usage in all protein-coding genes of the mitogenome of *S.calvus*.

The protein-coding regions spanned 11,407 bp, accounting for 64.26% of the length of the complete mitogenome of *S.calvus*. All PCGs, except for *ND6*, were transcribed on the plus strand. The predominant start codon for most PCGs was ATG, except for *COX1*, which utilized GTG as the start codon (Table 2). A detailed overview of the RSCU and codon distribution in the protein-coding genes of the mitogenome of *S.calvus* is provided. The codons CUA (L), CCU (P), and CUC (L) exhibited the highest frequency of occurrence (Fig. 2). A total of 1,257 SSRs were identified in the mitogenome, comprising 314 (24.98%) mono-, 529 (42.08%) di-, 301 (23.95%) tri-, 78 (6.21%) tetra-, 24 (1.91%) penta- and 11 (0.88%) hexanucleotide repeats. The ND5 gene contained the highest number of SSRs with 135 repeats (Table 3).

**Table 3.** Number of short sequence repeats in mitochondrial genome of *Sarcogypscalvus*. Abbreviations: MRS, monomeric repeated sequences; DRS, dinomeric repeated sequences; TriRS, trimeric repeated sequences; TetRS, tetrameric repeated sequences.

Region	MRS	DRS	TriRS	TetRS	Microsatellite sequences	Total
(unidentified region)	7	0	4	0	2	13
*ATP6*	7	18	14	3	3	45
*ATP8*	4	4	5	1	0	14
*COX1*	11	56	28	7	2	104
*COX2*	7	15	14	4	1	41
*COX3*	10	27	16	2	1	56
*CR1*	22	46	15	3	2	88
*CR2*	24	44	16	2	4	90
*CYTB*	18	37	16	6	3	80
*l*-*rRNA*	43	49	18	5	0	115
*ND1*	17	38	15	2	3	75
*ND2*	18	28	20	4	5	75
*ND3*	3	9	3	2	0	17
*ND4*	27	41	34	2	1	105
*ND4L*	1	10	3	2	0	16
*ND5*	32	53	41	7	2	135
*ND6*	14	10	12	5	5	46
*s-rRNA*	20	22	10	3	1	56
*trnA*	1	1	0	1	0	3
*trnC*	0	3	0	0	0	3
*trnD*	1	1	0	0	0	2
*trnE* (*uuc*)	2	1	1	0	0	4
*trnG*	2	5	2	0	0	9
*trnH* (*gug*)	1	1	0	2	0	4
*trnI*	0	4	0	0	0	4
*trnK*	1	2	4	0	0	7
*trnL*	1	3	0	0	0	4
*trnL* (*uag*)	1	0	0	0	0	1
*trnM*	2	0	2	0	0	4
*trnN*	1	0	0	1	0	2
*trnP* (*ugg*)	2	2	0	0	0	4
*trnQ*	2	3	2	0	0	7
*trnR*	0	1	0	1	0	2
*trnS*	0	1	1	2	0	4
*trnS* (*gcu*)	2	1	0	0	0	3
*trnT* (*ugu*)	2	4	0	0	0	6
*trnV*	0	1	3	2	0	6
*trnW*	1	2	0	0	0	3
*trnY*	4	0	0	0	0	4
Total	311	543	299	69	35	1257

**Figure 3.**
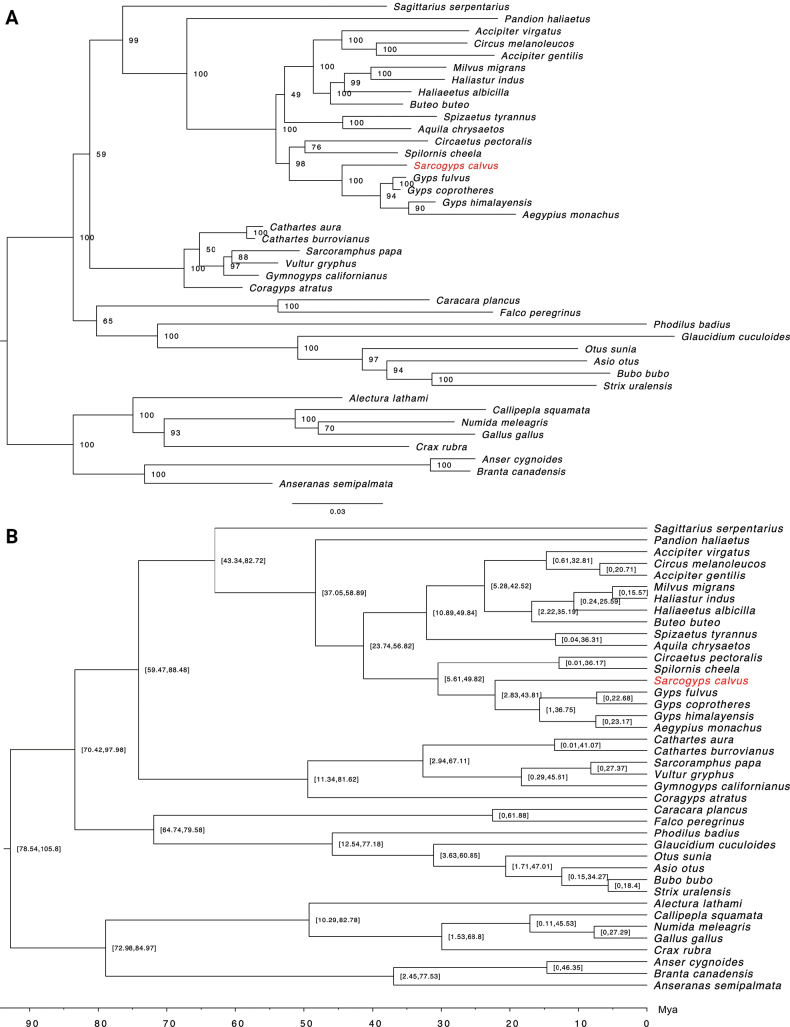
Maximum-likelihood phylogenetic trees based on amino acid alignments of 13 conserved protein-coding genes from the mitochondrial genomes of *Sarcogypscalvus* and 39 other bird species, with species from the orders Galliformes and Anseriformes used as outgroups **A** phylogram indicating bootstrap support values based on 1,000 replicates at each node **B** time calibrated phylogeny with the estimated minimum and maximum divergence times (millions of years ago, Mya) are indicated at each node. *Sarcogypscalvus* is in red.

The mitogenome of *S.calvus* was aligned with 39 previously published mitogenomes of bird species from the orders Accipitriformes, Cathartiformes, Falconiformes, Strigiformes, Anseriformes and Galliformes. Maximum likelihood phylogenies are illustrated in Fig. 3. The mitogenome of *S.calvus* was part of a clade formed by the tribe Gypini (*Gypsfulvus*, *Gypscoprotheres*, *Gypshimalayensis* and *Aegypiusmonachus*). Gypini formed a sister group with the serpent-eagles of the tribe Circaetini (*Spilornischeela* Latham, 1790 and *Circaetuspectoralis* Smith, 1829). Gypini and Circaetini formed the sister-group of a clade comprising the subfamilies Accipitrinae and Aquilinae. The subfamily Aquilinae included the species *Spizaetustyrannus* Wied, 1820 and *Aquilachrysaetos*, whereas the subfamily Accipitrinae included the tribe Accipitrini (*Accipitervirgatus* Temminck, 1822, *Accipitergentilis* and *Circusmelanoleucos* Forster, 1795) and the tribe Buteonini (*Milvusmigrans*, *Haliasturindus* Boddaert, 1783, *Haliaeetusalbicilla* and *Buteobuteo*) (Fig. 3A). The estimated evolutionary divergence time between *S.calvus* and its sister taxa, based on mitochondrial data, was approximately 22.2 million years ago (Mya) (95% highest posterior density (HPD): 2.8–43.8 Mya) (Fig. 3B). Fossil calibration constraints were applied to several groups, including Accipitriformes and Falconiformes, as well as Accipitriformes and Galliformes, among others. The estimated divergence times for these groups ranged from 6.2 to 101.5 Mya. Genetic distance analysis showed that the genetic distance between *S.calvus* and other Gypini ranged from 4.02% to 5.17%, while the distance between *S.calvus* and Cathartidae ranged from 10.90% to 12.26% (Suppl. material 1: table S1).

A total of 138 conserved amino acid substitution sites were observed between Old World vulture (Gypini) and New World vulture (Cathartidae) groups. The largest number of substitutions was found in the *ND5* gene (23 sites), whereas the smallest number occurred in *ATP6* (3 sites) (Suppl. material 1: table S2). Among these, 67 sites displayed substitutions between different amino acid chemical groups, with *ND1* showing the highest number of such substitutions (11 sites). Special case amino acid substitutions were found in *ATP8* (1 site), *COX1* (1 site), *COX2* (1 site), *CYTB* (2 sites), *ND1* (2 sites), *ND3* (2 sites), *ND4* (1 site), *ND4L* (1 site) and *ND6* (5 sites). Among these, Pro was the most frequently substituted amino acid, with 7 substitutions across *ATP8*, *CYTB*, *ND1*, *ND3* and *ND6*, followed by Cys (5 sites) and Gly (4 sites) (Table 4). In the Old World vulture clade, 43 amino acid substitution sites were identified between *S.calvus* and other Gypini. The largest number of substitutions was found in *CYTB* (9 sites), while the smallest number occurred in *COX1*, *COX2* and *ND4L* (1 site each) (Table 5). Unique amino acid chemical groups were found at 15 sites in *S.calvus*, with the largest number located in *ND5* (5 sites). Pro was the most frequently substituted amino acid in this group (3 sites in *ATP8*, *CYTB* and *ND4*), with Gly ranking second (1 site in *ND5*) (Table 5).

**Table 4.** Amino acid substitution with different side chain property between Old World vultures (OWVs) and New World vultures (NWVs).

Gene	Position	OWV	NWV	Side chain property	
				OWV	NWV
*ATP8*	7	A	N	Hydrophobic	Polar uncharged
	30	P	S	Special case	Polar uncharged
*COX1*	1	G	A	Special case	Hydrophobic
	3	S	F	Polar uncharged	Hydrophobic
*COX2*	4	H	N	Positive	Polar uncharged
	43	A	T	Hydrophobic	Polar uncharged
	90	N	D	Polar uncharged	Negative
	156	N	G	Polar uncharged	Special case
	161	A	S	Hydrophobic	Polar uncharged
	166	A	T	Hydrophobic	Polar uncharged
*COX3*	152	M	T	Hydrophobic	Polar uncharged
	224	N	K	Polar uncharged	Positive
*CYTB*	5	P	I	Special case	Hydrophobic
	376	M	T	Hydrophobic	Polar uncharged
	378	C	Y	Special case	Hydrophobic
	381	T	K	Polar uncharged	Positive
*ND1*	9	H	Y	Positive	Hydrophobic
	79	T	I	Polar uncharged	Hydrophobic
	81	M	T	Hydrophobic	Polar uncharged
	160	V	T	Hydrophobic	Polar uncharged
	171	A	T	Hydrophobic	Polar uncharged
	173	T	A	Polar uncharged	Hydrophobic
	189	T	A	Polar uncharged	Hydrophobic
	260	Q	E	Polar uncharged	Negative
	263	T	P	Polar uncharged	Special case
	312	I	T	Hydrophobic	Polar uncharged
	323	C	Y	Special case	Hydrophobic
*ND2*	5	T	A	Polar uncharged	Hydrophobic
	56	T	A	Polar uncharged	Hydrophobic
	65	T	A	Polar uncharged	Hydrophobic
	140	V	T	Hydrophobic	Polar uncharged
	185	A	S	Hydrophobic	Polar uncharged
	229	T	M	Polar uncharged	Hydrophobic
	299	H	Y	Positive	Hydrophobic
*ND3*	7	T	M	Polar uncharged	Hydrophobic
	19	I	T	Hydrophobic	Polar uncharged
	64	L	P	Hydrophobic	Special case
	77	T	P	Polar uncharged	Special case
*ND4*	8	T	M	Polar uncharged	Hydrophobic
	40	H	Q	Positive	Polar uncharged
	57	C	G	Special case	Special case
	63	S	A	Polar uncharged	Hydrophobic
	112	A	T	Hydrophobic	Polar uncharged
	170	H	Q	Positive	Polar uncharged
	171	I	T	Hydrophobic	Polar uncharged
	195	L	S	Hydrophobic	Polar uncharged
	201	M	T	Hydrophobic	Polar uncharged
*ND4L*	11	S	A	Polar uncharged	Hydrophobic
	16	C	S	Special case	Polar uncharged
	43	T	A	Polar uncharged	Hydrophobic
*ND5*	16	A	T	Hydrophobic	Polar uncharged
	61	S	M	Polar uncharged	Hydrophobic
	71	I	T	Hydrophobic	Polar uncharged
	332	T	M	Polar uncharged	Hydrophobic
	350	A	N	Hydrophobic	Polar uncharged
	382	M	T	Hydrophobic	Polar uncharged
	407	A	T	Hydrophobic	Polar uncharged
	438	M	T	Hydrophobic	Polar uncharged
	597	T	I	Polar uncharged	Hydrophobic
*ND6*	36	G	A	Special case	Hydrophobic
	50	P	S	Special case	Polar uncharged
	53	A	S	Hydrophobic	Polar uncharged
	73	C	S	Special case	Polar uncharged
	78	L	P	Hydrophobic	Special case
	107	E	G	Negative	Special case
	126	V	S	Hydrophobic	Polar uncharged
	140	W	R	Hydrophobic	Positive

**Table 5.** Amino acid substitution with different side chain property between Old World vultures (OWVs) and New World vultures (NWVs).

Gene	Position	OWV	NWV	Side chain property	
				OWV	NWV
*ATP6*	31	F	I	Hydrophobic	Hydrophobic
	83	I	I	Hydrophobic	Hydrophobic
	139	I	I	Hydrophobic	Hydrophobic
*ATP8*	24	I	I	Hydrophobic	Hydrophobic
	50	S	P	Polar uncharged	Special case
*COX1*	468	M	M	Hydrophobic	Hydrophobic
*COX2*	70	I	I	Hydrophobic	Hydrophobic
*CYTB*	15	I	I	Hydrophobic	Hydrophobic
	26	P	S	Special case	Polar uncharged
	47	L	L	Hydrophobic	Hydrophobic
	213	I	V	Hydrophobic	Hydrophobic
	220	P	P	Special case	Special case
	307	F	F	Hydrophobic	Hydrophobic
	310	K	K	Positive	Positive
	321	L	L	Hydrophobic	Hydrophobic
	370	T	L	Polar uncharged	Hydrophobic
*ND1*	15	S	S	Polar uncharged	Polar uncharged
	179	L	L	Hydrophobic	Hydrophobic
*ND2*	19	I	I	Hydrophobic	Hydrophobic
	22	S	S	Polar uncharged	Polar uncharged
	122	S	S	Polar uncharged	Polar uncharged
	325	T	T	Polar uncharged	Polar uncharged
	327	I	T	Hydrophobic	Polar uncharged
	335	I	L	Hydrophobic	Hydrophobic
*ND3*	1	I	I	Hydrophobic	Hydrophobic
	108	T	N	Hydrophobic	Polar uncharged
*ND4*	43	L	L	Hydrophobic	Hydrophobic
	90	A	T	Hydrophobic	Polar uncharged
	183	H	P	Positive	Special case
	263	T	T	Polar uncharged	Polar uncharged
	357	T	T	Polar uncharged	Polar uncharged
	394	I	I	Hydrophobic	Hydrophobic
	418	T	T	Polar uncharged	Polar uncharged
*ND4L*	73	T	T	Polar uncharged	Polar uncharged
*ND5*	30	T	T	Polar uncharged	Polar uncharged
	74	M	T	Hydrophobic	Polar uncharged
	291	T	T	Polar uncharged	Polar uncharged
	404	Y	Y	Hydrophobic	Hydrophobic
	434	E	G	Negative	Special case
	600	I	I	Hydrophobic	Hydrophobic
*ND6*	3	A	T	Hydrophobic	Polar uncharged
	142	A	A	Hydrophobic	Hydrophobic
	166	M	L	Hydrophobic	Hydrophobic

## ﻿Discussion

The present study has, for the first time, characterized the complete mitogenome of *S.calvus* and compared it with 39 other avian mitogenomes. The mitogenome of *S.calvus* included 13 PCGs, 22 tRNA genes, two rRNA genes and two putative CR regions, consistent with the mitogenomes of the *Gypshimalayensis* and *Aepygiuscinereus* (Li et al. 2015; Jiang et al. 2019). The total length of the PCG region of *S.calvus* was 11,407 base pairs (bp), which fell within the range observed in other members of Accipitriformes (11,377–11,920 bp). We observed that mitogenomes are subject to weaker translational selection compared to nuclear genomes (dos Reis et al. 2004). Regarding the GTG initiation codon of *COX1*, a previous study has reported the utilization of GTG as a start codon in the ribosomal protein L16 (rpl16) gene in some plant mitochondria (Bock et al. 1994). Additionally, RNA editing could also be a factor, as it has been observed in chicken mitochondria (Yokobori and Pääbo 1997). In the context of RSCU values, we identified AGG and AGA as the preferred stop codons in the mitogenome of *S.calvus*, with the RSCU values of 1.7 and 1.52, respectively. AGG has been identified as a stop codon for the NADH dehydrogenase 1 (*ND1*) and cytochrome c oxidase subunit 1(*COX1*) mitochondrial genes in the cinereous vulture and Himalayan griffon (Li et al. 2015; Jiang et al. 2019). Similarly, AGA has been shown to function as a stop codon for the NADH dehydrogenase subunit 3 (*NADH3*) and NADH dehydrogenase subunit 5 (*NADH5*) mitochondrial genes in the ostrich (Härlid et al. 1997). In contrast, UAA and UAG stop codons exhibited a negative bias, with RSCU values of 0.64 and 0.14, respectively. Additionally, we detected a negative bias against guanine at the third codon position across all 13 PCGs, consistent with findings in the cinereous vulture and Himalayan griffon (Li et al. 2015; Jiang et al. 2019).

Our results corroborate the position of *S.calvus* within the Old World vulture clade (Gypini), consistent with previous studies (Seibold and Helbig 1995; Lerner and Mindell 2005; Mindell et al. 2018; Khatri et al. 2019; Catanach et al. 2024). We estimated that *S.calvus* diverged from its sister clade (*Gyps* and *Aegypius*) approximately 22 Mya, while the divergence between Gypini and Cathartidae was estimated to have occurred around 74.1 Mya. This estimate closely aligns with previous findings (De Panis et al. 2021). Our analysis of amino acid substitutions, particularly those involving different chemical groups, suggests that these changes could potentially influence protein structure and function (Teng et al. 2010). Substitutions involving Cys, Pro, and Gly are particularly significant due to their unique roles in protein structure and stability. In this study, we observed transitions from Cys, which forms disulfide bonds critical for protein stability, to hydrophobic residues, potentially affecting protein folding and stability (Zavodszky et al. 2001; Alvares et al. 2013). Additionally, we detected changes involving Pro, known to restrict backbone flexibility, and Gly, which contributes to protein folding due to its small size, and may disrupt protein dynamics (Wilman et al. 2014; Senthil et al. 2019). We also noticed substitutions from hydrophobic to polar uncharged residues, such as Ser and Thr, across mitochondrial genes. These residues enhance hydrogen bonding and stability in aqueous environments, although transitions between similar residues (e.g., Ser to Thr) likely have minimal structural impact (Saeki et al. 2013). The observed amino acid changes may reflect functional adaptations and divergence within Gypini, with implications for mitochondrial function and the conservation of *S.calvus*. Future studies should explore these findings using structural modeling to better understand their impact.

## ﻿Conclusions

Our study documents the characteristics of the complete mitogenome of *S.calvus*. Phylogenetic analysis corroborated its evolutionary relationships within Accipitriformes. *S.calvus* was most closely related to a clade formed by *Aegypiusmonachus* and species of *Gyps*. Additionally, we identified conserved amino acid changes between Gypini and Cathartidae, as well as unique amino acid substitutions specific to the *S.calvus*. These findings enhance our understanding of the evolutionary history and functional genomics of this critically endangered species.

## ﻿Additional information

### Author ORCIDs

Sithichoke Tangphatsornruang https://orcid.org/0000-0003-2673-0012

Piroon Jenjaroenpun https://orcid.org/0000-0002-1555-401X

Thidathip Wongsurawat https://orcid.org/0000-0002-3659-2074

Vorasuk Shotelersuk https://orcid.org/0000-0002-1856-0589

Gunnaporn Suriyaphol https://orcid.org/0000-0002-8961-5970

### Data availability

All of the data that support the findings of this study are available in the main text or Supplementary Information.

## Supplementary materials

Supplementary material 1 Additional tables Data type pdf Explanation note **table S1.** Pairwise genetic distances of *Sarcogypscalvus* and related species. Abbreviations: OWV, Old World vulture; NWV, New World vulture. **table S2.** Conserved amino acid substitution between Old World vultures (OWV) and New World vultures (NWV). https://binary.pensoft.net/file/1302205This dataset is made available under the Open Database License (http://opendatacommons.org/licenses/odbl/1.0/). The Open Database License (ODbL) is a license agreement intended to allow users to freely share, modify, and use this Dataset while maintaining this same freedom for others, provided that the original source and author(s) are credited.Wannapol Buthasane, Sithichoke Tangphatsornruang, Piroon Jenjaroenpun, Thidathip Wongsurawat, Saowaphang Sanannu, Vorasuk Shotelersuk, Gunnaporn Suriyaphol
