# Supplementary material for: ﻿Complete mitogenome of the critically endangered Asian king vulture (Sarcogypscalvus) (Aves, Accipitriformes, Accipitridae): evolutionary insights and comparative analysis
Source: Zookeys. 2025 Apr 8;1234:47–65. doi: 10.3897/zookeys.1234.138722 (PMC12000817; doi:10.3897/zookeys.1234.138722)
Supplement: Supplementary material 1 — Additional tables [file zookeys-1234-047_article-138722__-s001.pdf]

Supplementary material 1

Table S1 Pairwise genetic distances of *Sarcogyps calvus* and related species

Abbreviations: OWV, Old World vulture; NWV, New World vulture

Compared species	Genetic distance from *S. calvus*	Order	Family	Clade
Gyps coprotheres	0.0402	Accipitriformes	Accipitridae	OWV
Gyps fulvus	0.0413	Accipitriformes	Accipitridae	OWV
Aegypius monachus	0.0498	Accipitriformes	Accipitridae	OWV
Gyps himalayensis	0.0517	Accipitriformes	Accipitridae	OWV
Spilornis cheela	0.0637	Accipitriformes	Accipitridae	serpent_eagles
Aquila chrysaetos	0.0693	Accipitriformes	Accipitridae	non_serpent_eagles
Buteo buteo	0.0698	Accipitriformes	Accipitridae	non_serpent_eagles
Circaetus pectoralis	0.0699	Accipitriformes	Accipitridae	serpent_eagles
Spizaetus tyrannus	0.0726	Accipitriformes	Accipitridae	non_serpent_eagles
Haliaeetus albicilla	0.0740	Accipitriformes	Accipitridae	non_serpent_eagles
Haliastur indus	0.0765	Accipitriformes	Accipitridae	non_serpent_eagles
Milvus migrans	0.0770	Accipitriformes	Accipitridae	non_serpent_eagles
Accipiter virgatus	0.0877	Accipitriformes	Accipitridae	non_serpent_eagles
Circus melanoleucos	0.0910	Accipitriformes	Accipitridae	non_serpent_eagles
Accipiter gentilis	0.0927	Accipitriformes	Accipitridae	non_serpent_eagles
Coragyps atratus	0.1090	Accipitriformes	Cathartidae	NWV
Gymnogyps californianus	0.1099	Accipitriformes	Cathartidae	NWV
Vultur gryphus	0.1109	Accipitriformes	Cathartidae	NWV
Sarcoramphus papa	0.1160	Accipitriformes	Cathartidae	NWV

Cathartes burrovianus	0.1211	Accipitriformes	Cathartidae	NWV
Cathartes aura	0.1226	Accipitriformes	Cathartidae	NWV
Sagittarius serpentarius	0.1366	Accipitriformes	Sagittariidae	secretarybird
Pandion haliaetus	0.1386	Accipitriformes	Pandionidae	osprey
Anseranas semipalmata	0.1539	Anseriformes	Anseranatidae	waterfowl
Caracara plancus	0.1562	Falconiformes	Falconidae	falcons
Alectura lathami	0.1659	Galliformes	Megapodiidae	landfowl
Crax rubra	0.1732	Galliformes	Cracidae	landfowl
Numida meleagris	0.1765	Galliformes	Numididae	landfowl
Falco peregrinus	0.1773	Falconiformes	Falconidae	falcons
Gallus gallus	0.1806	Galliformes	Phasianidae	landfowl
Asio otus	0.1831	Strigiformes	Strigidae	owls
Bubo bubo	0.1853	Strigiformes	Strigidae	owls
Strix uralensis	0.1868	Strigiformes	Strigidae	owls
Otus sunia	0.1884	Strigiformes	Strigidae	owls
Callila squamata	0.1894	Galliformes	Odontophoridae	landfowl
Phodilus badius	0.1912	Strigiformes	Tytonidae	owls
Branta canadensis	0.1922	Anseriformes	Anatidae	waterfowl
Anser cygnoides	0.1941	Anseriformes	Anatidae	waterfowl
Glaucidium cuculoides	0.2120	Strigiformes	Strigidae	owls

Table S2 Conserved amino acid substitution between Old World vultures (OWV) and New World vultures (NWV)

Gene	Position	OWV	NWV	Side chain property	
				OWV	NWV
ATP6	63	S	N	Polar uncharged	Polar uncharged
	123	T	S	Polar uncharged	Polar uncharged
	205	I	V	Hydrophobic	Hydrophobic
ATP8	7	A	N	Hydrophobic	Polar uncharged
	11	Y	F	Hydrophobic	Hydrophobic
	20	L	F	Hydrophobic	Hydrophobic
	30	P	S	Special case	Polar uncharged
COX1	1	G	A	Special case	Hydrophobic
	3	S	F	Polar uncharged	Hydrophobic
	401	L	F	Hydrophobic	Hydrophobic
	404	F	Y	Hydrophobic	Hydrophobic
	493	V	I	Hydrophobic	Hydrophobic
COX2	4	H	N	Positive	Polar uncharged
	7	L	F	Hydrophobic	Hydrophobic

	43	A	T	Hydrophobic	Polar uncharged
	60	I	V	Hydrophobic	Hydrophobic
	74	M	L	Hydrophobic	Hydrophobic
	90	N	D	Polar uncharged	Negative
	122	V	I	Hydrophobic	Hydrophobic
	126	D	E	Negative	Negative
	141	M	V	Hydrophobic	Hydrophobic
	156	N	G	Polar uncharged	Special case
	161	A	S	Hydrophobic	Polar uncharged
	166	A	T	Hydrophobic	Polar uncharged
	190	V	I	Hydrophobic	Hydrophobic
	225	M	L	Hydrophobic	Hydrophobic
COX3	152	M	T	Hydrophobic	Polar uncharged
	171	F	L	Hydrophobic	Hydrophobic
	179	N	T	Polar uncharged	Polar uncharged
	224	N	K	Polar uncharged	Positive
CYTB	5	P	I	Special case	Hydrophobic
	30	I	A	Hydrophobic	Hydrophobic
	61	S	T	Polar uncharged	Polar uncharged
	226	S	P	Polar uncharged	Polar uncharged
	305	I	M	Hydrophobic	Hydrophobic
	326	Y	F	Hydrophobic	Hydrophobic
	339	I	V	Hydrophobic	Hydrophobic
	357	I	L	Hydrophobic	Hydrophobic
	365	V	I	Hydrophobic	Hydrophobic
	376	M	T	Hydrophobic	Polar uncharged
	378	C	Y	Special case	Hydrophobic
	381	T	K	Polar uncharged	Positive
ND1	9	H	Y	Positive	Hydrophobic
	68	V	I	Hydrophobic	Hydrophobic
	79	T	I	Polar uncharged	Hydrophobic
	81	M	T	Hydrophobic	Polar uncharged
	90	L	I	Hydrophobic	Hydrophobic
	160	V	T	Hydrophobic	Polar uncharged
	171	A	T	Hydrophobic	Polar uncharged
	173	T	A	Polar uncharged	Hydrophobic
	189	T	A	Polar uncharged	Hydrophobic
	260	Q	E	Polar uncharged	Negative
	263	T	P	Polar uncharged	Special case
	312	I	T	Hydrophobic	Polar uncharged
	321	V	L	Hydrophobic	Hydrophobic
	323	C	Y	Special case	Hydrophobic
ND2	5	T	A	Polar uncharged	Hydrophobic
	14	I	L	Hydrophobic	Hydrophobic
	24	T	N	Polar uncharged	Polar uncharged
	35	L	I	Hydrophobic	Hydrophobic

	43	F	L	Hydrophobic	Hydrophobic
	56	T	A	Polar uncharged	Hydrophobic
	65	T	A	Polar uncharged	Hydrophobic
	70	L	V	Hydrophobic	Hydrophobic
	76	T	S	Polar uncharged	Polar uncharged
	140	V	T	Hydrophobic	Polar uncharged
	175	L	V	Hydrophobic	Hydrophobic
	185	A	S	Hydrophobic	Polar uncharged
	221	S	T	Polar uncharged	Polar uncharged
	229	T	M	Polar uncharged	Hydrophobic
	237	S	T	Polar uncharged	Polar uncharged
	299	H	Y	Positive	Hydrophobic
	341	L	I	Hydrophobic	Hydrophobic
ND3	4	V	I	Hydrophobic	Hydrophobic
	7	T	M	Polar uncharged	Hydrophobic
	17	I	L	Hydrophobic	Hydrophobic
	19	I	T	Hydrophobic	Polar uncharged
	23	L	F	Hydrophobic	Hydrophobic
	64	L	P	Hydrophobic	Special case
	77	T	P	Polar uncharged	Special case
ND4	8	T	M	Polar uncharged	Hydrophobic
	40	H	Q	Positive	Polar uncharged
	57	C	G	Special case	Special case
	63	S	A	Polar uncharged	Hydrophobic
	78	L	M	Hydrophobic	Hydrophobic
	105	L	F	Hydrophobic	Hydrophobic
	107	L	I	Hydrophobic	Hydrophobic
	112	A	T	Hydrophobic	Polar uncharged
	121	T	S	Polar uncharged	Polar uncharged
	138	S	N	Polar uncharged	Polar uncharged
	170	H	Q	Positive	Polar uncharged
	171	I	T	Hydrophobic	Polar uncharged
	195	L	S	Hydrophobic	Polar uncharged
	201	M	T	Hydrophobic	Polar uncharged
	250	I	M	Hydrophobic	Hydrophobic
	333	T	N	Polar uncharged	Polar uncharged
	343	L	I	Hydrophobic	Hydrophobic
	426	L	I	Hydrophobic	Hydrophobic
ND4L	11	S	A	Polar uncharged	Hydrophobic
	16	C	S	Special case	Polar uncharged
	43	T	A	Polar uncharged	Hydrophobic
	62	F	L	Hydrophobic	Hydrophobic
ND5	16	A	T	Hydrophobic	Polar uncharged
	18	L	I	Hydrophobic	Hydrophobic
	61	S	M	Polar uncharged	Hydrophobic
	66	S	T	Polar uncharged	Polar uncharged

	71	I	T	Hydrophobic	Polar uncharged
	84	I	M	Hydrophobic	Hydrophobic
	90	I	L	Hydrophobic	Hydrophobic
	107	S	T	Polar uncharged	Polar uncharged
	122	F	Y	Hydrophobic	Hydrophobic
	175	I	L	Hydrophobic	Hydrophobic
	190	Y	W	Hydrophobic	Hydrophobic
	193	S	T	Polar uncharged	Polar uncharged
	328	F	L	Hydrophobic	Hydrophobic
	332	T	M	Polar uncharged	Hydrophobic
	350	A	N	Hydrophobic	Polar uncharged
	372	T	S	Polar uncharged	Polar uncharged
	382	M	T	Hydrophobic	Polar uncharged
	407	A	T	Hydrophobic	Polar uncharged
	438	M	T	Hydrophobic	Polar uncharged
	451	L	I	Hydrophobic	Hydrophobic
	460	M	L	Hydrophobic	Hydrophobic
	592	T	S	Polar uncharged	Polar uncharged
	597	T	I	Polar uncharged	Hydrophobic
ND6	8	L	F	Hydrophobic	Hydrophobic
	11	A	V	Hydrophobic	Hydrophobic
	36	G	A	Special case	Hydrophobic
	45	V	L	Hydrophobic	Hydrophobic
	50	P	S	Special case	Polar uncharged
	53	A	S	Hydrophobic	Polar uncharged
	73	C	S	Special case	Polar uncharged
	78	L	P	Hydrophobic	Special case
	95	M	L	Hydrophobic	Hydrophobic
	107	E	G	Negative	Special case
	114	A	W	Hydrophobic	Hydrophobic
	126	V	S	Hydrophobic	Polar uncharged
	140	W	R	Hydrophobic	Positive
